# Dexamethasone suppresses the differentiation of stem Leydig cells in rats in vitro

**DOI:** 10.1186/s40360-019-0312-z

**Published:** 2019-05-27

**Authors:** Jingwei Zhang, Guanghui Hu, Bisheng Huang, Dong Zhuo, Yujie Xu, Huitao Li, Xiangcheng Zhan, Ren-Shan Ge, Yunfei Xu

**Affiliations:** 1 Department of Urology, Shanghai Tenth People’s Hospital, Tongji University, Shanghai, China; 2 Department of Urology, Yijishan Hospital, Wannan Medical College, Wuhu, Anhui China; 3 Center of Scientific Research, the Second Affiliated Hospital and Yuying Children’s Hospital of Wenzhou Medical University, Wenzhou, Zhejiang China; 4 Department of Urology, Shanghai Tenth People’s Hospital, Nanjing Medical University, Nanjing, Jiangsu China

**Keywords:** Glucocorticoid, Dexamethasone, Stem Leydig cells, Proliferation, Differentiation, Testosterone

## Abstract

**Background:**

It is an established fact that excess of glucocorticoids could cause the harmful effects, such as suppression on the male reproduction. Although glucocorticoids pharmacologically inhibit the Leydig cell function, their roles in Leydig cell development are unclear. Therefore, the present study was designed to investigate effects of synthetic glucocorticoid dexamethasone (DEX) on rat stem Leydig cell proliferation and differentiation.

**Methods:**

Male Sprague-Dawley rats received a single intraperitoneal injection of 75 mg/kg EDS to eliminate Leydig cells and an in vitro culture system of the seminiferous tubules was established from Leydig cell-depleted testis. Using basal medium and Leydig cell differentiation-inducing medium (LIM) in the culture system, we examined the effects of DEX (0–100 nM) on the proliferation and differentiation of the stem Leydig cells in vitro*,* respectively.

**Results:**

Results showed that LIM is a good agent to induce stem Leydig cell differentiation into Leydig cells that produce testosterone in vitro*.* DEX inhibited the differentiation of stem Leydig cells by reducing the expression levels of *Cyp17a1* and *Scarb1* and that NR3C1 antagonist RU38486 reversed the DEX-mediated effects. However, DEX are not involved with the proliferation of stem Leydig cells.

**Conclusions:**

DEX suppressed the differentiation of rat Leydig cells in vitro and glucocorticoid-induced effects acted through NR3C1. This suppression partially targets on *Cyp17a1* and *Scarb1* gene expression.

## Background

Glucocorticoids play roles in many physiological functions [1]. Through binding to the glucocorticoid receptor (NR3C1, encoded by *Nr3c1*), glucocorticoids function in the level of cells. NR3C1 is known to be a member of the nuclear steroid receptor superfamily and a ligand-dependent transcription factor that acts as a regulator in the expressions of glucocorticoid-responsive genes [2]. Endogenous glucocorticoids are cortisol (the predominant glucocorticoid in the human) or corticosterone (the main glucocorticoid in the rat). Glucocorticoids can pharmacologically inhibit many physiological functions such as the suppression of immune system, brain, and reproduction [1, 3]. Clinically, the potent synthetic glucocorticoids such as dexamethasone (DEX) are widely used as the drugs to treat symptoms of inflammation and immune disorders such as systemic lupus erythematosus. However, the long-term use of DEX could cause many side effects.

Indeed, glucocorticoids can suppress the male reproduction [1]. One of the targets by glucocorticoids is the Leydig cell in the testis. Leydig cells are the main cells to secrete testosterone, which is necessary for the onset of male puberty and spermatogenesis and the maintenance of the secondary sexual characteristics [4]. Leydig cells contain NR3C1 and glucocorticoids suppress testosterone biosynthesis mediated by NR3C1 [5–7]. However, the effects of glucocorticoids on the pubertal development of Leydig cells are unclear. Clinically, it has been demonstrated that the children with the treatment of > 400 mg/kg cumulative dose of corticosteroids in systemic lupus erythematosus had a delay of puberty [8].

In the present study, a Sprague-Dawley rat Leydig cell development was used to address the effects of DEX on Leydig cell development. The pubertal development of rat Leydig cells in vivo can be conceptually divided into four stages: stem, progenitor, immature, and adult Leydig cells [4]. This development of Leydig cells can be mimicked in vitro by the induction of stem Leydig cells into the Leydig cell lineage on the surface of the seminiferous tubules after 21 days of culture in the presence of Insulin-Transferrin-Selenite (ITS), luteinizing hormone (LH) and lithium (LI) together [9, 10]. In this model, Leydig cells were eliminated by a drug called ethane dimethane sulfonate (EDS) and the Leydig cell-free tubules were cultured [9, 10]. The stem Leydig cells were located on the surface of the seminiferous tubules and they can either enter the proliferative status or commit to the Leydig cells in the presence of different growth factors [11]. Stem Leydig cells can commit to the Leydig cell lineage by expressing androgen-biosynthetic enzymes, including CYP11A1 (encoded by *Cyp11a1*), HSD3B1 (encoded by *Hsd3b1*), CYP17A1 (encoded by *Cyp17a1*), and HSD17B3 (encoded by *Hsd17b3*), SRD5A1 (encoded by *Srd5a1*). In addition, LHCGR (encoded by *Lhcgr*), SCARB1 (encoded by *Scarb1*) and StAR (encoded by *Star*) also play essential roles in Leydig cell development. In the present study, we examined the effects of DEX on the proliferation and differentiation of the stem Leydig cells in vitro.

## Methods

### Chemicals

DEX and RU38486 (NR3C1 antagonist) were purchased from Sigma-Aldrich (MO, USA). EDS was purchased from Pterosaur Biotech Co. (Zhejiang, China).

### Animals

Male Sprague-Dawley rats were purchased from Shanghai Laboratory Animal Co. Ltd. (Shanghai, China). Rats were adjusted in a 12 h dark/light cycle at 23 ± 2 °C and 45 to 55% relative humidity for a week, given ad libitum accesse to water and food. At the time of experiment, the median weight of rats was 523 g (399 g ~ 607 g) and the median age of rats was 91 days (86 days ~ 95 days). EDS was dissolved in dimethyl sulfoxide and sterile water (1,3, v/v) and these rats received a single intraperitoneal injection of 75 mg/kg EDS to eliminate Leydig cells according to the previous study [12]. All animal procedures were approved by the Institutional Animal Care and Use Committee of Tongji University and were performed in accordance with the National Research Council (US) Committee Guide for the Care and Use of Laboratory Animals.

### Seminiferous tubule isolation and culture

After EDS treatment, rats were housed for additional 7 days to deplete all Leydig cells [13]. At this time, the rats were killed by asphyxiation with CO_2_ of progressively increasing concentration (100% eventually) and underwent bilateral orchidectomy via a 2- to 3-cm abdominal incision, then testes were collected immediately and decapsulated. Seminiferous tubules were mechanically separated from the interstitium. Tubule fragments of equal total length (about 3 cm) were placed randomly in each well of 12-well plates. Basal medium (BM) was used for culturing the seminiferous tubules at 34 °C and 5% CO_2_, which was a mixture of DMEM/F-12 plus an equal volume of Medium 199 (pH 7.2), sodium bicarbonate (2.2 mg/mL), bovine serum albumin (1 mg/mL), and penicillin/streptomycin (100 U/mL and 100 μg/mL), respectively. For proliferation investigation of stem Leydig cells, seminiferous tubules were cultured without or with DEX (1, 10, and 100 nM). The concentrations of DEX were selected based on its inhibition on steroidogenesis in rat progenitor Leydig cells [14].

Leydig cell medium was selected based on the treatment of BM in presence of 5 mM ITS, 5 ng/ml LH, 5 mM LI, or ITS + LH + LI [9, 15]. The Leydig cell differentiation-inducing medium (LIM) was constructed by containing 5 mM ITS, 5 ng/ml LH, and 5 mM LI. The seminiferous tubules in LIM were cultured for 21 days on which stem Leydig cells fully differentiate into the Leydig cell lineage [9, 15]. To investigate the effects, we added DEX and/or RU38486 (a NR3C1 blocker) of different concentrations to this LIM on the basis of the previous studies [5, 16].

Media were collected and frozen (− 20 °C) for testosterone assay. Tubules were immediately fixed for morphological study, total RNA extraction, and total protein extraction. Duplicated wells were used at each time point, and each experiment was replicated at least three times.

### Medium testosterone analysis

The IMMULITE® 2000 Immunoassay System and Total Testosterone Kit from Siemens Healthcare Diagnostics Products Limited (Gwynedd, UK) was used to detect medium testosterone concentrations, according to the manufacturer’s instructions. The immunochemiluminometric assay was able to detect as low as 0.2 ng/ml testosterone with 5.75 and 7.53% of intra- and inter-assay CVs.

### 3β-Hydroxysteroid dehydrogenase enzymatic staining to label Leydig cells

After the culture in BM or LIM for 21 days, seminiferous tubules tubules were air-dried and stained to evaluate HSD3B1 activity of Leydig cells as previously described [17]. At a minimum, air-dried tubules were incubated with 0.4 mM etiocholanolone as the steroid substrate and NAD^+^ as a cofactor for 30 min. A staining solution without 0.4 mM etiocholanolone was used for non-specific staining. Then, tubules were washed with PBS and fixed using 4% paraformaldehyde for 30 min at room temperature. The tubules were visualized in a microscope.

### EDU incorporation into stem Leydig cells

Stem Leydig cells reside on the surface and their proliferation was measured by the Click-iT® EDU (EDU) Alexa Fluor® 488 Imaging Kit (Life Technologies, OR, USA) following the manufacturer’s instructions and the EDU incorporation into cells was performed as previously described [11]. In our previous study, we demonstrated that EDU-positive cells are located outside myoid cells [11]. Briefly, the seminiferous tubules were isolated and cultured in the BM without or with DEX for the additional 5 days. Following this culture, tubules were incubated in 2 ml of 1:1000 diluted EDU for 24 h and then were washed twice with PBS buffer containing 3% bovine serum albumin. Tubules were subsequently fixed in 4% paraformaldehyde for 30 min at room temperature and washed twice. Then, tubules were incubated with reaction solution for 45 min and washed again, protected against exposure to light. Finally, tubules mounted on a slide were visualized using a fluorescence microscope (Olympus, Tokyo, Japan). By the aid of Image Pro Plus 6.0 software (Media Cybernetics, Inc., MD, USA), EDU-positive cells were calculated by the total surface area of the seminiferous tubules.

### Cell immunofluorescence

Seminiferous tubules cultured in the LIM for 21 days were embedded in 2% argose gel and cross sections (10 μm) were cut. The sections were fixed in 4% paraformaldehyde for 30 min at room temperature and washed with Ca^2+^ and Mg^2+^ free HBSS (0.5% BSA). Then sections were incubated with mouse monoclonal antibody against α-smooth muscle actin (α-SMA, Abcam, ab7817, 1:200) and rabbit monoclonal antibody against CYP17A1 (Abcam, ab125022, 1:200) for 60 min. Following the primary antibody incubation, sections were incubated with conjugated secondary antibody (Donkey anti-Mouse IgG (H + L) Highly Cross-Adsorbed Secondary Antibody, Alexa Fluor® 594, Invitrogen, A-21203, 1:500) & (Donkey Anti-Rabbit IgG (H + L) Dylight 488, Bioworld, BS10018, 1:100) for 30 min. Peritubular myoid cells were labeled by α-SMA and Leydig cells were labeled by CYP17A1. DAPI (Abcam Trading Shanghai Company, Shanghai, China) was used as a counterstain. The sections were visualized under a fluorescence microscope (Olympus, Tokyo, Japan) and CYP17A1-positive cells were counted and calculated. We counted five cross sections and averaged them for each experiment.

### Leydig cell isolation

A complete description of the cell isolation procedure in the Leydig cell lineage has been described [18]. The stem Leydig cells were isolated according to immunoselection method by positively selecting platelet-derived growth factor receptor α (PDGFRA) positive cells and negatively selecting LHCGR-negative cells from forty 6-day-old male rat testis [19]. The purity of stem Leydig cells was about 99% after immunofluroscent staining of PDGFRA using anti-PDGFRA antibody. Progenitor Leydig cells were purified from forty 21-day-old rat testes [18]. Testes were decapsulated and dispersed in medium 199 plus 0.25 mg/ml collagenase (Boehringer Mannheim Biochemicals, Indianapolis, IN) by a shaking 34 °C water bath (75 rpm) for 10 min. The separated cells were harvested and filtered through two layers of nylon mesh (100 μm). Then the cells were centrifuged at 250×g and resuspended in 55% isotonic Percoll. Density gradient centrifugation was subsequently conducted at 25000×g for 45 min at 4 °C, and the progenitor Leydig cell fraction was collected between densities of 1.064 and 1.070 g/ml. After washing with HBSS, the cells were centrifuged at 250×g and resuspended in phenol red-free medium (DMEM-Ham’s F-12, Sigma, St. Louis, MO) containing 1 mg/ml BSA. Immature Leydig cells were isolated from eighteen 35-day-old rat testes [18]. Testes were perfused with 1 mg/ml collagenase in medium 199 via the testicular artery before decapsulation [18]. Other procedure was similar to progenitor Leydig cell isolation. The immature Leydig cell fraction was collected from the Percoll gradient between densities of 1.07 and 1.088 g/ml. Adult Leydig cells were purified from six 90-day-old rat testes according to the method of Klinefelter et al. [20]. Before the Percoll density gradient centrifugation, collagenase dispersed interstitial cells were elutriated in the Beckman JE-6B elutriation chamber (Palo Alto, CA) at a flow rate of 16 ml/min at 2000 rpm, after which adult Leydig cells were collected from the Percoll gradient between densities of 1.07 and 1.09 g/ml. Purities of Leydig cell fractions for progenitor, immature and adult Leydig cells were determined by HSD3B1 staining as previously described [21]. The purities of progenitor, immature, and adult Leydig cells were more than 95%. Four isolations were performed for each cell types. The Leydig cells of all cell types were collected for RNA extraction.

### Real-time quantitative polymerase chain reaction (qPCR)

Total RNAs were extracted from cells in the Leydig cell lineage and the seminiferous tubules cultured in the LIM for 21 days using Trizol® Reagent (Life Technologies, CA, USA) according to the manufacturer’s instruction. The Leydig cell genes and corresponding primers with at least one span intron were used as described previously [22–25], including *Nr3c1*, *Lhcgr*, *Scarb1*, *Star*, *Cyp11a1*, *Hsd3b1*, *Cyp17a1*, *Hsd17b3*, *Srd5a1*. *Rps16* was used as an internal control gene for the relative mRNA levels. Reactions were performed and the cDNA was synthesized using Reverse Transcription System (Promega, WI, USA). The qPCR was performed in a 25-μl reaction volume using SYBR Green detection system (Bio-Rad Laboratories, Inc., CA) and Light Cycler®480 SYBR Green I Master (Roche Diagnostics, IN, USA). Reactions were run for up to 40 cycles and the melting curves were routinely checked afterward. The data were analyzed using the Ct value and the standard curve method as previously described [26].

### Western blotting

Seminiferous tubules were cultured in the LIM for 21 days and radioimmuno-precipitation assay buffer (Beyotime Biotechnology, Shanghai, China) was used to isolate total proteins. An Enhanced BCA Protein Assay Kit (Beyotime Biotechnology, Shanghai, China) was used to determine the protein concentrations. 50 μg of proteins were loaded onto 10% polyacrylamide gels containing sodium dodecyl sulfate and transferred onto the nitrocellulose membranes. The membranes were blocked with 5% non-fat milk for 2 h and were incubated overnight at 4 °C with primary antibodies: anti-SCARB1 (MultiSciences, 70-ab1967–050, 1:1500), anti-CYP17A1 (MultiSciences, 70-ab1766–050, 1:1000), and anti-β-actin (ACTB, Cell Signaling Technology, 4970, 1:1000). After washing, the membranes were incubated with HRP-conjugated anti-rabbit IgG secondary antibody (MultiSciences, 70-GAR0072, 1:2000) for 2 h at room temperature and washed again. Membranes were processed with Western Bright® ECL (Advansta, CA, USA) and the intensity of immunoreactive bands were analyzed with Image Lab software 3.0 (Bio-Rad Laboratories, Inc., CA).

### Statistical analysis

Values are expressed as mean ± SEM, and data were analyzed by the GraphPad Prism 6 (GraphPad Software Inc., CA). Multiple groups were performed by one-way ANOVA followed by ad hoc Tukey’s comparison of all columns compared with the control column. Before ANOVA was used for statistical analysis, the normal distribution and variance homogeneity were confirmed. Mean value comparisons between 2 groups were performed by student t test. Before student t test was used for statistical analysis, the normal distribution is confirmed. Differences were considered significant at *P* < 0.05.

## Results

### LIM can induce stem Leydig cell into the Leydig cell lineage

**Fig. 1:**
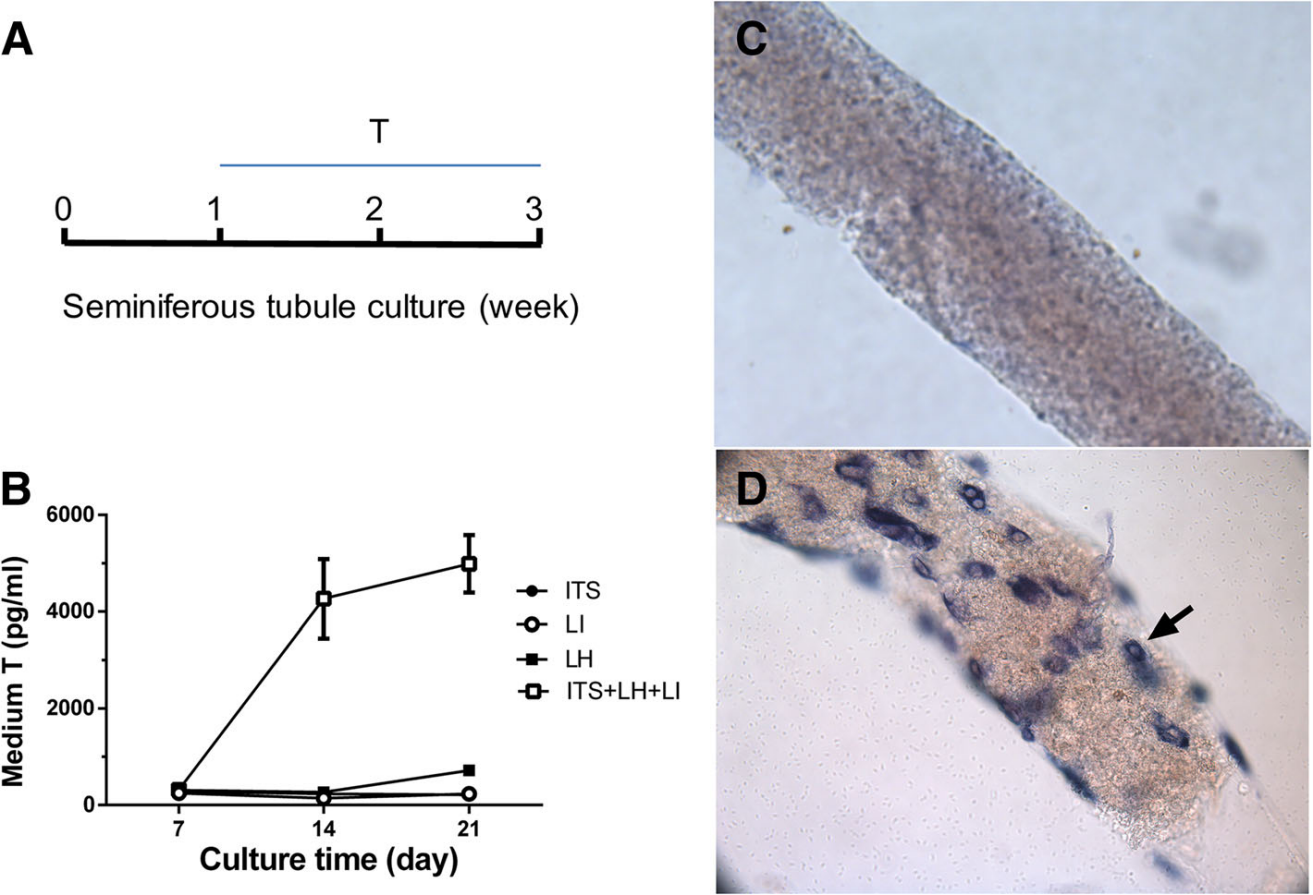
An in vitro culture system for differentiating stem Leydig cells on the surface of seminiferous tubule into Leydig cell lineage. Panel **a**-**b**: The testosterone production by seminiferous tubules in the BM contained ITS 5 mM, LH 5 ng/ml, LI 5 mM, or ITS 5 mM + LH 5 ng/ml + LI 5 mM (the LIM). Mean ± SEM, *n* = 4–8. Panel **c-d**: Seminiferous tubules were stained with HSD3B1 and Leydig cells were indicated by black arrows. Panel: (**c**) Seminiferous tubule after 21 days of culture in the BM (no Leydig cells were observed); (**d**) Leydig cells were observed with HSD3B1 staining (cells indicated by black arrow on the surface of a seminiferous tubule) after 21 days of culture in the LIM

To investigate the condition of in vitro differentiation of stem Leydig cells into the Leydig cell lineage, we cultured the seminiferous tubules in BM (control), or BM containing the following agents: ITS, LH, LI, or ITS + LH + LI. As shown in Fig. 1a-b, only ITS + LH + LI robustly induced testosterone production during week 2–3. Compared to the BM (Fig. 1c), which had no Leydig cells formed on the surface of the seminiferous tubules, ITS + LH + LI-treated tubules had Leydig cells with 18 ± 1 Leydig cells/1000μm^2^ (Fig. 1d). This indicates that LIM is a good agent to induce stem Leydig cell differentiation into Leydig cells that produce testosterone in vitro*.*

### DEX blocks stem Leydig cell differentiation

**Fig. 2:**
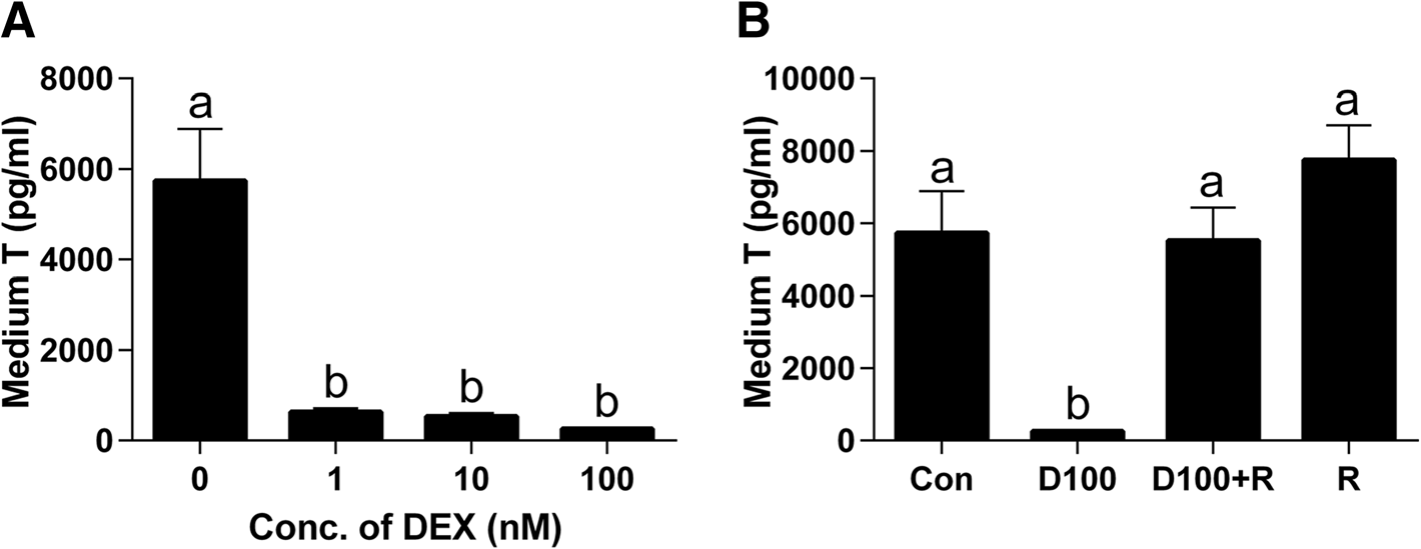
Effects of DEX on the differentiation of stem Leydig cells. Seminiferous tubules were cultured in the LIM in the presence of DEX alone or with RU38486 or RU38486 alone for 21 days, medium testosterone (T) levels were measured (*n* = 4–8). Panel: (**a**) DEX (0–100 nM); (**b**) DEX alone or with RU38486 or RU38486 alone. D100 = DEX 100 nM and R = RU38486 1 μM. The data were presented as the mean ± SEM. Identical letters showed no significant difference between two groups at *P* < 0.05

**Fig. 3:**
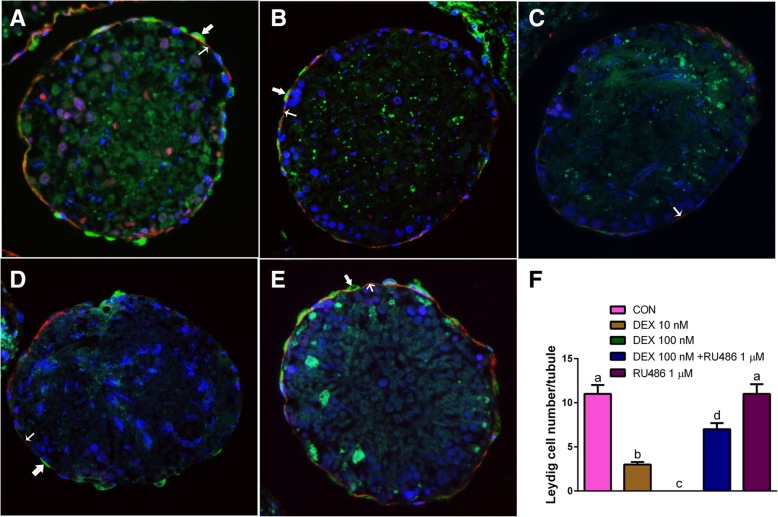
DEX reduces the formation of Leydig cells. Panel **a**-**f**: Immunofluorescent staining of tubule section after DEX alone or with RU38486 or RU38486 alone treatment in the LIM for 21 days. Cross sections with CYP17A1 staining (green color, bold arrow) showed the formation of Leydig cells. α-Smooth muscle actin staining (red color, light arrow) showed the myoid cells, which surround the seminiferous tubules. CYP17A1 positive cells were outside the α-smooth muscle actin positive cells, indicating that they were differentiated from the stem Leydig cells on the surface of the tubule. Panel: (**a**) DEX 0 nM; (**b**) DEX 10 nM; (**c**) DEX 100 nM; (**d**) DEX 100 nM + RU38486 1 μM; (**e**) RU38486 1 μM; (**f**) Quantitative data of Leydig cell number per cross section (*n* = 3). The data were presented as the mean ± SEM. Identical letters showed no significant difference between two groups at *P* < 0.05

To induce the stem Leydig cells to differentiate into Leydig cells, the seminiferous tubules were cultured in LIM for consecutive 21 days, thus producing testosterone as above. When the seminiferous tubules were cultured for 21 days in LIM plus various concentrations of DEX, DEX concentration-dependently decreased medium testosterone levels (Fig. 2a). This signifies that DEX blocks Leydig cell development/steroidogenesis. Then, we further examined whether the effects of DEX were antagonized by RU38486, a NR3C1 antagonist. Indeed, RU38486 1 μM alone did not affect the Leydig cell development/steroidogenesis, but it reversed DEX-induced testosterone suppression (Fig. 2b). This indicates that DEX blocks the Leydig cell development/steroidogenesis via the NR3C1. We stained the Leydig cells after 21-day culture in LIM by CYP17A1, the biomarker of Leydig cells. Leydig cells appeared on the surface of seminiferous tubule (11 ± 3 Leydig cells/tubule in the cross section, Fig. 3a). When cultured with 10 nM DEX, CYP17A-positive Leydig cell number formed was remarkably reduced (Fig. 3b). When 100 nM DEX was used, almost no Leydig cells were formed (Fig. 3c). When 100 nM DEX and RU38486 1 μM in combination were used, DEX-mediated suppression of the formation of CYP17A1-positive Leydig cells were reversed (Fig. 3d), although RU38486 alone did not affect the formation of Leydig cells (Fig. 3e). The quantitative data was listed on Fig. 3f. This further confirmed that DEX inhibited the formation of CYP17A1-positive Leydig cells and that RU38486 reversed the DEX-mediated effects.

### DEX down-regulates Leydig cell-specific genes and protein expressions

**Fig. 4:**
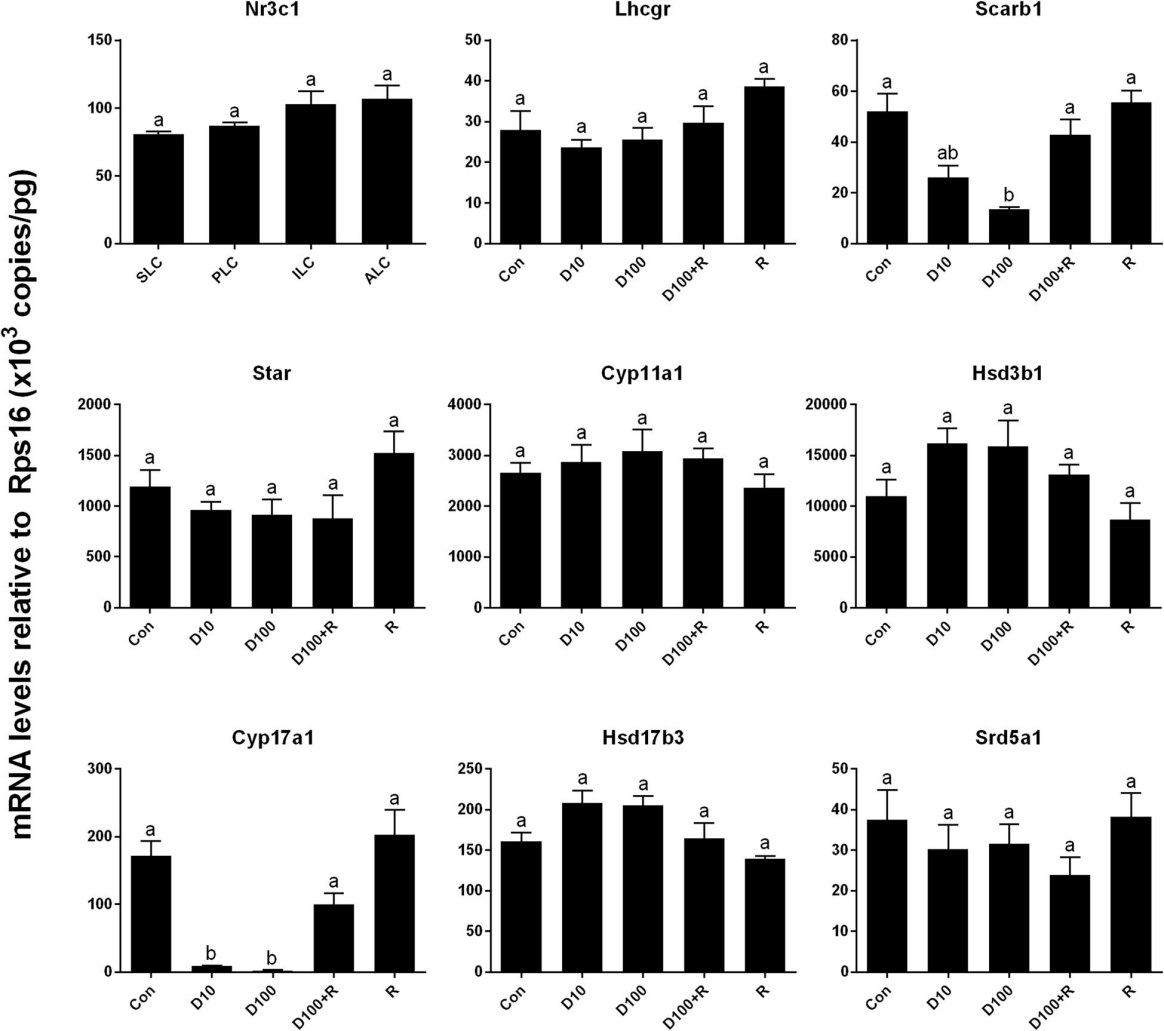
The expressions of *Nr3c1* in the Leydig cell lineage and the effects of DEX on Leydig cell gene expressions. Stem (SLC), progenitor (PLC), immature (ILC), adult (ALC) Leydig cells were isolated from 6-, 21-, 35-, and 90-day-old rat testes, respectively. Seminiferous tubules were cultured in the LIM in the presence of DEX alone or with RU38486 or RU38486 alone for 21 days, then the qPCR measured mRNA levels of Leydig cell-specific genes (n = 4–6). D10 = DEX 10 nM, D100 = DEX 100 nM, R = RU38486 1 μM. The data are presented as the mean ± SEM. Identical letters showed no significant difference between two groups at *P* < 0.05

**Fig. 5:**
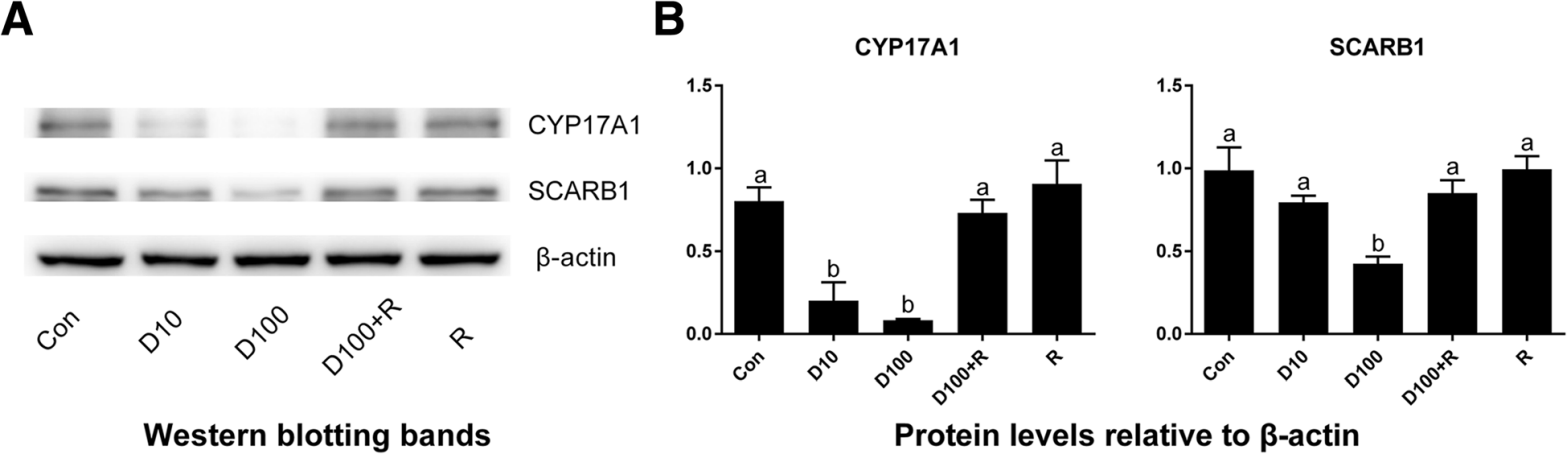
DEX down-regulates protein levels of CYP17A1 and SCARB1. Seminiferous tubules were cultured in the LIM in the presence of DEX alone or with RU38486 or RU38486 alone for 21 days. Panel **a**: Western blot measured the proteins levels of CYP17A1 and SCARB1. Panel **b**: Quantitative data of protein levels (n = 3). D10 = DEX 10 nM, D100 = DEX 100 nM, R = RU38486 1 μM. The data are presented as the mean ± SEM. Identical letters showed no significant difference between two groups at *P* < 0.05

In the previous study, we used regular PCR and found that *Nr3c1* was detected in progenitor, immature, and adult Leydig cells [5]. In the present study, we also included stem Leydig cells and used qPCR to measure *Nr3c1* levels and we found that *Nr3c1* was present in all Leydig cell lineage cells (since the negative control showed no signal, data not shown), including stem Leydig cells, further supporting that stem Leydig cells are the target of DEX. We examined the effects of DEX on the expression levels of Leydig cell-specific genes (Fig. 4). Statistically, we found 10 and 100 nM DEX significantly down-regulated the mRNA levels of the steroidogenic enzyme gene *Cyp17a1* (encoding CYP17A1) and scavenger receptor class B member 1 gene *Scarb1* (encoding SCARB1). However, DEX did not affect other 6 mRNA levels. In parallel, DEX also down-regulated CYP17A1 and SCARB1 protein levels (Fig. 5). RU38486 reversed DEX-mediated effects. These results indicate that DEX inhibits the differentiation of Leydig cells by partially reducing the expression levels of *Cyp17a1* and *Scarb1.*

### DEX does not influence stem Leydig cell proliferation

**Fig. 6:**
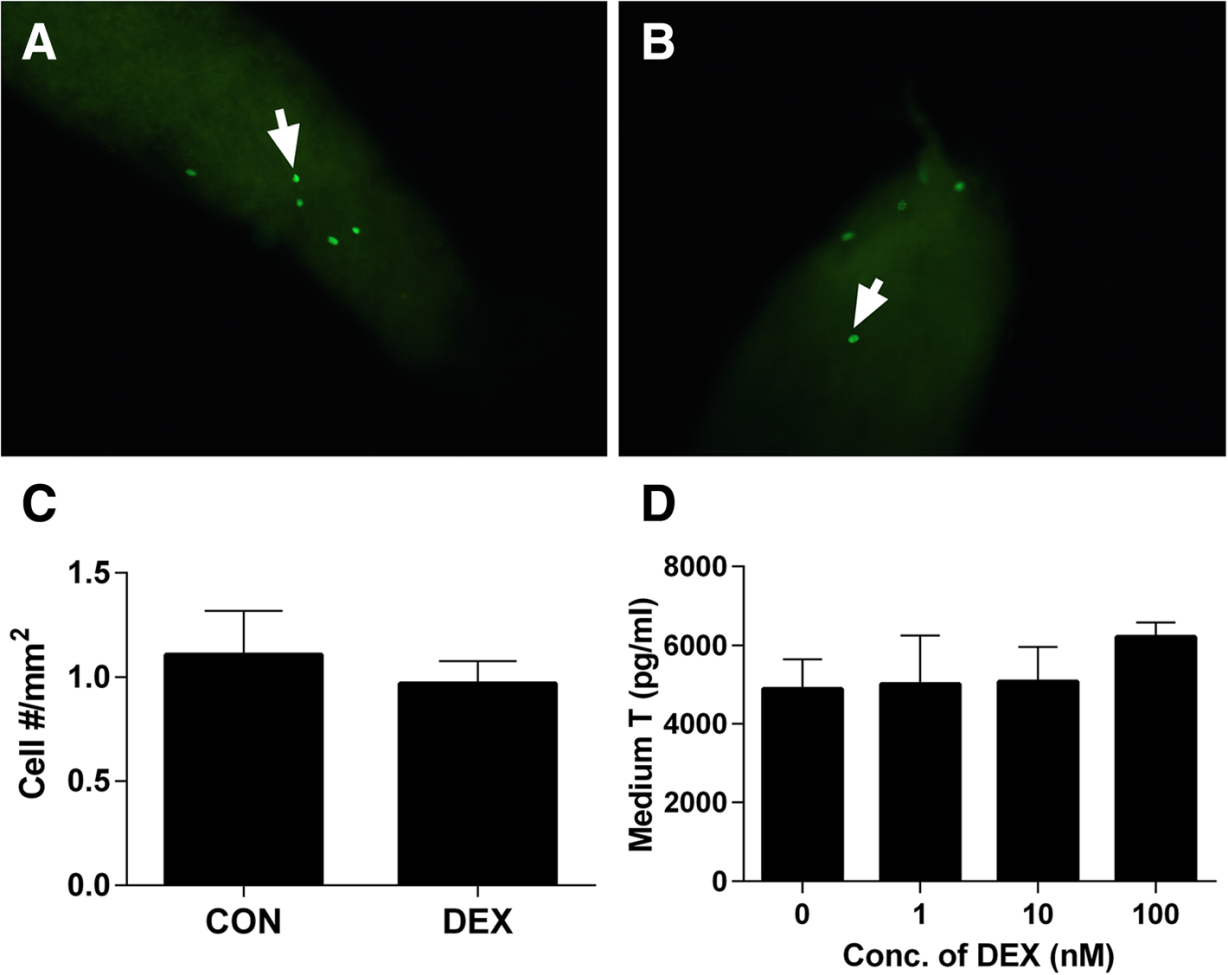
DEX does not affect stem Leydig cell proliferation. Panel **a**-**c**: EDU-labeled stem Leydig cells (green color, white arrow) afte treatment in the BM for 5 days. Panel: (**a**) Control; (**b**) DEX 100 nM; (**c**) EDU-labeled stem Leydig cell numbers (*n* = 6). Panel D: Medium testosterone (T) levels after seminiferous tubules were treated with various concentrations of DEX (0–100 nM) in the BM for 7 days and then these tubules were switched to the LIM for additional 14 days (n = 4–8). In Panel **c** and **d**, the data were presented as the mean ± SEM and there was no significant difference when compared to control group at *P* < 0.05

To explore the probalble effects of DEX on the proliferation of stem Leydig cells, we used an in vitro seminiferous tubule culture system. In this system, isolated seminiferous tubules were cultured for 5 days without or with 1–100 nM DEX in the BM. Then, EDU incorporation into stem Leydig cells on the surface of seminiferous tubules were observed and EDU-positive cells were located outside myoid cells [15]. As shown in Fig. 6a-c, in the BM, the EDU incorporative rate into the stem Leydig cells was 1.11 ± 0.21 cells/mm^2^ and DEX treatment did not affect the EDU incorporation rate into stem Leydig cells. This suggests that DEX are not involved with the proliferation of stem Leydig cells.

In order to further investigate whether DEX can affect stem Leydig cells pool, we cultured the seminiferous tubules in the BM without or with various concentrations of DEX (1–100 nM) for 7 days. To induce these stem Leydig cells into the Leydig cells, the tubules were transferred to the LIM for extra 14 days. Testosterone levels in the medium were not significantly affected when compared to control (Fig. 6d), confirming that DEX did not increase stem Leydig cell number.

## Discussion

Endogenous glucocorticoid, cortisol or corticosterone, are required for the development in fetal tissues and organs. However, both acute and chronic stress experiments have shown that high glucocorticoids inhibited testosterone secretion [27]. This effect is due specifically to the increased circulating glucocorticoid levels in the blood and action via NR3C1 because ACTH treatment in adrenalectomized animals fails to replicate these findings [28]. In this study, we found that glucocorticoids also targeted stem Leydig cells to inhibit their differentiation.

In the present study, we demonstrated that stem Leydig cells on the surface of the Leydig cell-depleted seminiferous tubules in the medium containing ITS, LH, and LI could be induced into testosterone-producing Leydig cells in vitro. This system is very useful for testing the chemicals like DEX to regulate stem Leydig cell differentiation and proliferation without the interference of hypothalamus-pituitary secreted hormones as described [11]. In the present study, we found that NR3C1 was expressed in stem, progenitor, immature, and adult Leydig cells (Fig. 4). Our previous study also demonstrated that although the expression levels of *Nr3c1* were not changed, the binding sites of NR3C1 were significantly increased during later stage of development from immature to adult Leydig cells [29]. In this system, we found that DEX suppressed the differentiation of stem Leydig cells in vitro and glucocorticoid-induced effects acted through NR3C1.

The present study demonstrated that DEX suppressed the differentiation of stem Leydig cells in vitro and glucocorticoid-induced effects acted through down-regulating the expression levels of *Cyp17a1* and *Scarb1*. The down-regulation of these two proteins could cause the reduction of testosterone synthesis. Apparently, DEX acts via binding to NR3C1, since RU38486, a NR3C1 antagonist, reversed DEX-mediated suppression of *Cyp17a1* and *Scarb1* expressions (Fig. 4) and testosterone levels (Fig. 2). Lower level of *Cyp17a1* and its protein after DEX treatment were due to the absence of CYP17A1-positive Leydig cells (Fig. 3). CYP17A1 is among the first three important steroidogenic enzyme genes during the initial development of stem into progenitor Leydig cells [30]. Although the exact mechanism of DEX to down-regulate *Cyp17a1* in the Leydig cell lineage is not clear, research using adult-derived bovine steroidogenic cells has shown the competition between COUP-TFII and NR5A1 for binding to an overlapping response element in the promoter region of gene encoding *Cyp17a1* to inhibit NR5A1-induced *Cyp17a1* expression [31]. NR5A1 is a critical ligand-free nuclear receptor transcription factor for promoting Leydig cell development. NR5A1 can bind to the promoters of many Leydig cell genes including *Cyp17a1* [32]. Null mutation of NR5A1 caused Leydig cell agenesis [33]. Forced expression of NR5A1 can even convert stem cells or fibroblasts into steroidogenic Leydig cell-like cells by transcriptionally promoting the expression of steroidogenic enzymes including *Cyp17a1* [34, 35]. Apparently, the exposure to DEX in rats during the prenatal period significantly increased the COUP-TFII expression in fetal Leydig cells thus down-regulating *Cyp17a1* expression and inhibiting testosterone production [32]. Interestingly, DEX did not down-regulate other steroidogenic enzymes such as *Cyp11a1*, *Hsd3b1*, and *Hsd17b3*. This indicates that DEX partially blocks the differentiation of stem Leydig cells. How DEX can affect several steroidogenesis-related genes without affect others requires further investigation. Maybe, the DEX-mediated effects depend on the Leydig cell stages. For example, DEX inhibited steroidogenesis of progenitor Leydig cells by suppressing of *Star* and *Hsd3b1* via a glucocorticoid-mediated mechanism [16]. Besides, glucocorticoid-induced inhibition of testosterone biosynthesis occurred through the suppression of *Cyp11a1*, *Hsd3b1*, and *Cyp17a1* gene expression in adult Leydig cells. [7, 36–38]. However, the inhibition on steroidogenic enzyme gene expressions was happened during a short-term culture [7, 36–38]. For example, a 6-h immobilization stress significantly increased serum glucocorticoid level but down-regulated *Lhcgr*, *Star*, *Scarb1*, *Cyp11a1*, *Cyp17a1*, and *Hsd17b3* and reduced serum testosterone level [25]. However, in the present study, we treated the stem Leydig cells during a 21-day period and the effect of DEX was chronic.

Therefore, our study explored the mechanism of DEX to regulate rat Leydig cell development in vitro.

## Conclusions

DEX inhibits the differentiation of stem Leydig cells into Leydig cell lineage via NR3C1. This suppression partially targets on *Cyp17a1* and *Scarb1* gene expressions.

## Abbreviations

BM: Basal medium
CYP11A1: Cytochrome P450 cholesterol side-chain cleavage enzyme
CYP17A1: Cytochrome 17α-hydroxylase/20-lyase
DEX: Dexamethasone
EDS: Ethane dimethane sulfonate
HSD17B3: 17β-hydroxysteroid dehydrogenase 3
HSD3B1: 3β-hydroxysteroid dehydrogenase 1
ITS: Insulin-Transferrin-Selenite
LH: Luteinizing hormone
LHCGR: LH receptor
LI: Lithium
LIM: Leydig cell differentiation-inducing medium
PDGFRA: Platelet-derived growth factor receptor α
qPCR: Real-time quantitative polymerase chain reaction
SCARB1: High-density lipoprotein receptor
SRD5A1: Steroid 5α-reductase 1
StAR: Steroidogenic acute regulatory protein
α-SMA: α-smooth muscle actin
